# Application of water based drilling clay-nanoparticles in heat transfer of fractional Maxwell fluid over an infinite flat surface

**DOI:** 10.1038/s41598-021-98066-w

**Published:** 2021-09-22

**Authors:** Muhammad Imran Asjad, Rizwan Ali, Azhar Iqbal, Taseer Muhammad, Yu-Ming Chu

**Affiliations:** 1 Department of Mathematics, University of Management and Technology, Lahore, Pakistan; 2 Department of Mathematics, Dawood University of Engineering and Technology, Karachi, 74800 Pakistan; 3 Department of Mathematics, College of Sciences, King Khalid University, Abha, 61413 Saudi Arabia; 4 Department of Mathematics, Huzhou University, Huzhou, 313000 People’s Republic of China

**Keywords:** Applied mathematics, Chemical engineering, Mechanical engineering

## Abstract

In the present paper, unsteady free convection flow of Maxwell fluid containing clay-nanoparticles is investigated. These particles are hanging in water, engine oil and kerosene. The values for nanofluids based on the Maxwell-Garnett and Brinkman models for effective thermal conductivity and viscosity are calculated numerically. The integer order governing equations are being extended to the novel non-integer order fractional derivative. Analytical solutions of temperature and velocity for Maxwell fluid are build using Laplace transform technique and expressed in such a way that they clearly satisfied the boundary conditions. To see the impact of different flow parameters on the velocity, we have drawn some graphs. As a result, we have seen that the fractional model is superior in narrate the decay property of field variables. Some limiting solutions are obtained and compared with the latest existing literature. Moreover, significant results can be observed for clay nanoparticles with different base fluids.

## Introduction

Since the last decades, in various disciplines, fractional calculus theory has brought greater attention for the researchers. In fact, it was found that use of fractional derivatives is very helpful in modifying many process related to thermal transport processes, engineering sciences, circuit analysis, Biotechnology and signal processing. There are so many other applications related to heat and mass transfer and fluid dynamics can be perceived in the references^1–5^. Different books related to fractional derivatives were written by Jagdev et al.^6^, Kolade and Atangana^7^ and Baleanu et al.^8^ and they also discuss the application of fractional derivatives and also about their operators.

In literature different concepts related to fractional derivatives can be find out. In physics fractional derivative plays an important role in designing different phenomena. Yet, it was mentioned in Caputo and Fabrizio^9^, that different circumstances belonging to material heterogeneities cannot be well-modeled using fractional derivatives introduced by Riemann-Liouville or Caputo. Due to this fact, new fractional derivative related to non-singular kernel were introduced by Caputo and Fabrizio^9^. It was important that, rather than power law and exponential decay function the kernel Mittag- Leffler function is more general. Therefore, both Riemann-Liouville and Caputo-Fabrizio are special cases of Atangana-Baleanu fractional operators^10–13^. In applied sciences, some new kinds of derivatives exist which are known as fractal derivative. Therefore there was a need to redefined the concept of expressing velocity in fractal media for example, scaling time in fractal $(x, t^{\alpha })$. Baleanu et al.^14^ in 2020 introduced a new fractional operator using power law and are called hybrid fractional derivatives. This derivative is linear combination of constant proportional and Caputo type fractional derivative.

Ali^15^ investigated the Atangana-Baleanu derivative with a novel approach. Circuits with fractional derivatives were developed by Hammouch and Mekkaoui^16^ and they also discussed its behavioral dynamics. It is known that for description of price of opinion can be given by using Time Fractional Black Scholes Equation (TFBSE) with a time derivative of real order. The investigation of heat dissipation in transmission line of electrical circuit is given in Abro et al.^17^. An analysis of generalized Jeffery nanofluid in a rotating frame with non-singular fractional derivative is given in Ali et al.^18^. The behavior realted to heat transfer in different model with singular and non-singular is given in articles^19–22^.

To control the entropy in the flow of heat is one of the main concerns of the industrial sectors. Since the problem had a large amplitude so a lot of researchers pay attention to solve it. They use different methods for different fluids to enhance the thermal conductivity^23^. Bejan et al. has came to the conclusion that viscous dissipation, heat transfer, mass transfer and chemical reaction are the main reasons are entropy enhancement in thermal systems^24–27^. To overcome the problem the first meaningful contribution was made by Choi, when he gave the idea of nanofluids^28^. This was the revolution in the flow of heat and has given the answers to the many unsolved problems. He just added nanosized particles of different type to solve the problem of entropy. Entropy in magnetohydrodynamics (MHD) by having exact analysis is investigated by Khan et al.^29^. A definition regarding the Bejan number which is useful to predict the power of magnetic field and entropy of fluid friction due to heat transfer is given by Awed^30,31^. Saouli and Aiboud-Saouli^32^ has used an inclined plate and liquid film to analyzed entropy generation. Mahmud et al.^33^ reported the experiment with added magnetic field influence. Selimefendigil et al.^34^ has explored entropy for natural convection flow of a nanofluid. ^35–39^ made a useful contribution in this regard. ^40,41^ has discussed that how can nanofluids be used in solar energy system and evaporation. Brownian motion and thermophoresis effect in heat transfer was analyzed by Buongiorno^42^. Some valuable studies have been done in this regards by Biglarian et al.^43^, Mosayebidorcheh et al.^44^, Pourmehran et al.^45^, Rahimi-Gorji et al.^46^, Tesfai et al.^47^, Wu and Zhao^48^, Khan^49^, Sheikholeslami and Bhatti^50^, and Abdelsalam and Bhatti^51,52^.

The two-dimensional magnetohydrodynamics (MHD) flow of Casson fluid and heat transfer find out by Hamid et al.^53^. Mainly we focus the study is to survey the linear thermal radiation influence on dual solutions for both the steady and unsteady flow of Casson fluid under the effect of uniform magnetic field. The blood flow connecting nanoparticles through porous blood vessels in the occurence of magnetic field with the help of collocation and least squares techniques inspected by Usman et al.^54^. Blood is a non-Newtonian fluid having nanoparticles which are used for different models to find the viscosity of the nanofluids. Hamid et al.^55^ disscussed the unsteady MHD flow of Williamson nanofluid between the permeable channel with heat source/sink. The effect of molybdenum disulfide ($MoS_2$) nanoparticles forms on circling flow of nanofluid along an elastic stretched sheet. This nanofluid flow is measured in the existance of magnetic things, thermal radiation and variable thermal conductivity. Hamid et al.^56^ discussed the different types of nanoparticles like Platelet, cylindrical and brick forms. Usman et al.^57^ evaluate the flow of ethylene glycol and water based copper (Cu) nanoparticles between two squeezed parallel disks.

Newly, the industrialists are interested theoretically and experimentally studying the advancement of nanofluids. The industrialists are elaborated to find thermophysical properties (heat capacitance, thermal conductivity, density, electrical conductivity, thermal expansion, and viscosity) of unlike nanoparticles and base fluids using several procedures^58^ because the next generations fluid is a nanofluid for heat transport which can deal additional thermal presentation in various industrial sectors like power generation, transportation, hyperthermia and air conditioning.

Inspiriting from the above-discussed literature, this study aims to focus on the applications of nanofluid in the drilling process. Different type of nanofluid is used in drilling activities such as oil-based drilling mud (OBM), water-based drilling mud. For this purpose we have used the Polymers nanoparticles and clay nanoparticles. It has been observed that for stability in temperature, least value of torque, prevention of fluid loss, to control the rheological possessions for scrubbing the hole and to filter the quality of cake, clay nanoparticles helps a lot. Khan et al.^59^ has used three different base fluids to clean water. Nisar et al.^60^ has studied the entropy of clay nanoparticles.

The main purpose of this paper is to extend the idea of Imran et al.^61^ in which analytical solutions are obtained for viscous fluid. They used the Laplace transform method to obtain the solutions for temperature and velocity fields respectively. For the moment there is no such results regarding Maxwell fluid containing clay nanoparticles therefore, we have applied the most recent hybrid fractional operator for a Maxwell fluid of caly-water base nanofluids over an infinite vertical surface moving with constant velocity and obtained solutions with Laplace transform method. Some limiting solutions are also obtained and justified through graphical comparison and presented in the graphical section. Thermophysical properties of nanomaterials are defined in Table 1.

**Table 1:** Thermophysical properties of nanofluids^59^.

Material	Symbol	$\rho (\frac{1}{m^{3}} \times kg)$	$C_{p} (\frac{1}{kg K} \times J)$	K$(\frac{1}{m K} \times W)$	$\frac{\beta }{10^{5}} (\frac{1}{K})$	$\text {Pr}$
Clay	Nanoparticles	6320	531.8	76.5	1.80	–
Water	$H_2O$	997	4179	0.613	21	6.2
Kerosene oil	KO	783	2090	0.145	99	21
Engine oil	EO	884	1910	0.114	70	500

## Mathematical formulation and solution

**Figure 1:**
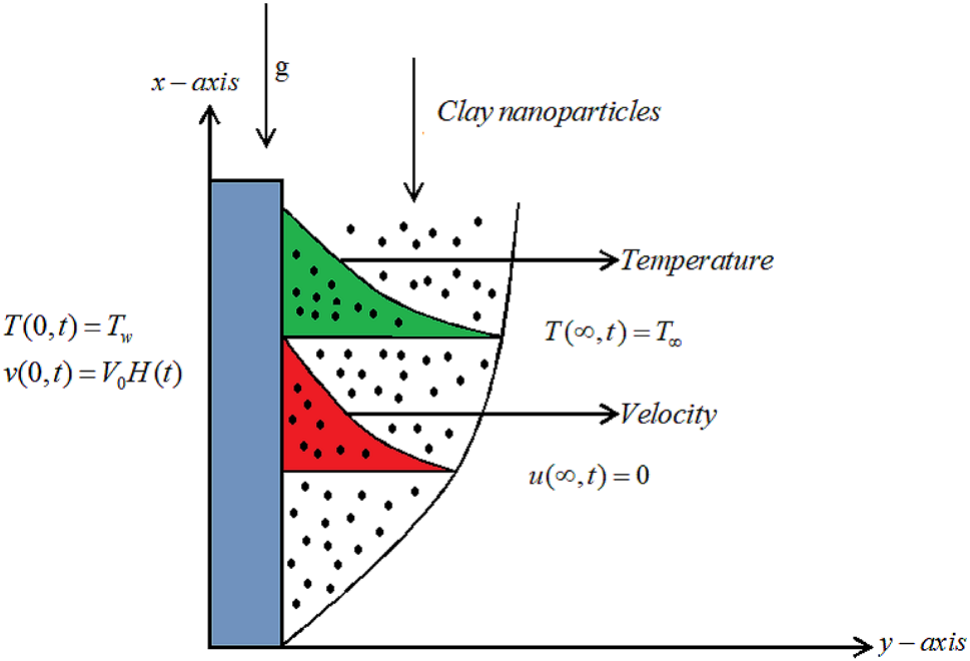
Geometry of the problem.

Let water, kerosene and engine oils are base fluids to carve-up in the flow of clay nanoparticles. The flow of the fluid is in the region $y_{1}>0$, close to a heated flat vertical plate. The plate is normal to y-axis and is fixed. In the beginning it is assumed that the fluid is at rest on the plate having surrounding temperature $T_{\infty }$. This ambient temperature changes from $T_{\infty }$ to $T_{w}$ in no time causing the motion in the plate with velocity $V_{0}$ forcing the fluid to move in *x*-direction as shown in Fig. 1. The governing equations are given as follows^62–65^.

Continuity Eq.^66^

1 $$\begin{aligned} \frac{\partial \rho }{\partial t} + \nabla (\rho v) = 0, \end{aligned}$$

where $\rho$ is the density of fluid, $\nabla$ is the divergence operator and *v* is the velocity of fluid. For incompressible fluid

2 $$\begin{aligned} \nabla . v = 0. \end{aligned}$$

Cauchy stress tensor for Maxwell model of the form^67^

3 $$\begin{aligned} T = -pI + S, \ \ \ S + \lambda _1 \frac{\delta S}{\delta t} = \mu A_1, \end{aligned}$$

where -p is the pressure, I is the identity matrix, $\mu$ is the viscosity, and $A_1$ is the first Rivlin-Eriksen tensor defined as

4 $$\begin{aligned}&A_1 = \nabla v + \nabla v^{T}, \end{aligned}$$

5 $$\begin{aligned}&\frac{\delta s}{\delta t} = \frac{D S}{D t} - L S - S L^{T}. \end{aligned}$$

where $\frac{D}{Dt}$ is the material time derivative, and *L* is gradient of the velocity.

Fractional stress tensor for Maxwell fluid with constant proportional Caputo time fractional derivative^14^

6 $$\begin{aligned} \tau + \lambda ^{\alpha }_{1} \frac{\partial ^{\alpha }\tau }{\partial t^{\alpha }} = \mu _{nf} \frac{\partial v}{\partial y}, \ \ \ 0<y<h, \ \ t>0 \end{aligned}$$

where $\tau = S_{xy}$ is the non zero component of extra stress tensor and $\frac{\partial \alpha }{\partial t^{\alpha }}$ is the constant proportional Caputo derivative of non integer order $\alpha$. The innovative fractional derivative is defined and given in^14^.

7 $$\begin{aligned} ^{CPC}D^{\alpha }_{t}h(t)=[\Gamma (1-\alpha )]^{-1}\int _{0}^{t}\frac{\left( L_{1}(\alpha )h(\tau )+L_{o}(\alpha ){\acute{h}}(\tau )\right) }{(t-\tau )^{\alpha }}d\tau ,\ \ 0<\alpha <1. \end{aligned}$$

The Laplace transform of constant proportional Caputo is given as^14^

8 $$\begin{aligned} {\L }\left\{ ^{CPC}D^{\alpha }_{t}h(t)\right\} =\left\{ \frac{L_{1}(\alpha )}{s}+L_{o}(\alpha )\right\} s^{\alpha }-L_{o}(\alpha )s^{\alpha -1}h(0). \end{aligned}$$

Navier–Stokes Eq.^66^

9 $$\begin{aligned} \rho _nf\left( \frac{\partial v}{\partial t}+v.(\nabla v)\right) = -\nabla p + \mu \frac{\partial \tau }{\partial y_1}. \end{aligned}$$

Multiplying Eq. (9) by $\left( 1 + \lambda ^{\alpha }_{1} \frac{\partial ^{\alpha }}{\partial t^{\alpha }} \right)$

10 $$\begin{aligned}&\rho _nf\left( \frac{\partial v}{\partial t}+v.(\nabla v)\right) \left( 1 + \lambda ^{\alpha }_{1} \frac{\partial ^{\alpha }}{\partial t^{\alpha }} \right) = (-\nabla p)\left( 1 + \lambda ^{\alpha }_{1} \frac{\partial ^{\alpha }}{\partial t^{\alpha }} \right) + \mu _nf \frac{\partial \tau }{\partial y_1}\left( 1 + \lambda ^{\alpha }_{1} \frac{\partial ^{\alpha }}{\partial t^{\alpha }} \right) \nonumber \\&\quad +g(\rho \beta _{T})_{nf}(T(y_{1}, t)-T_{\infty })\left( 1 + \lambda ^{\alpha }_{1} \frac{\partial ^{\alpha }}{\partial t^{\alpha }} \right) , \end{aligned}$$

In the absence of pressure gradient and convective term, and by using Eq. (6) into Eq. (10), we have

11 $$\begin{aligned} \rho _{nf}\frac{\partial v(y_{1}, t)}{\partial t}+ \rho _{nf} \lambda _{1}^{\alpha } \frac{\partial ^{\alpha +1}v(y_1,t)}{\partial t^{\alpha +1}}=\mu _{nf}\frac{\partial ^{2}v(y_{1}, t)}{\partial y_{1}^{2}}+ \left( 1+\lambda ^\alpha _{1} \frac{\partial ^\alpha }{\partial t^\alpha }\right) g(\rho \beta _{T})_{nf}(T(y_{1}, t)-T_{\infty }), \end{aligned}$$

Constitutive relation for thermal flux is given as

12 $$\begin{aligned} (\rho C_{p})_{nf}\frac{\partial T(y_{1},t)}{\partial t}= - \frac{\partial q(y_1,t)}{\partial y_1}, \end{aligned}$$

In order to find the fractional energy equation, applying the operator $\left( 1 + \lambda ^{\beta }_{1} \frac{\partial ^{\beta }}{\partial t^{\beta }}\right)$ on both sides of Eq. (10)^63,64^,

13 $$\begin{aligned} (\rho C_{p})_{nf} \left( 1 + \lambda ^{\beta }_{1} \frac{\partial ^{\beta }}{\partial t^{\beta }}\right) \frac{\partial T(y_{1},t)}{\partial t} = - \frac{\partial }{\partial y_1} \left( 1 + \lambda ^{\beta }_{1} \frac{\partial ^{\beta }}{\partial t^{\beta }}\right) q(y_1, t), \end{aligned}$$

Generalization of fractional Cattaneo’s law^68^

14 $$\begin{aligned} \left( 1 + \lambda ^{\beta }_{1} \frac{\partial ^{\beta }}{\partial t^{\beta }}\right) q(y_{1},t) = - K_{nf} \frac{\partial T(y_{1},t)}{\partial y_1}, \end{aligned}$$

Using Eq. (14) into Eq. (13), We have

15 $$\begin{aligned} (\rho C_{p})_{nf} \frac{\partial T(y_{1},t)}{\partial t} + (\rho C_{p})_{nf} \lambda ^{\beta }_{1} \frac{\partial ^{\beta +1}T(y_1,t)}{\partial t^{\beta +1}} = K_{nf} \frac{\partial ^{2}T(y_{1}, t)}{\partial y_{1}^{2}}, \end{aligned}$$

where $v = v(y_{1}, t)$, $T = T(y_{1},t)$, $\lambda _1$, *q*, $\rho _{nf}$, $\mu _{nf}$, $\beta _{T}$, *g*, $(\rho c_{p})_{nf}$, $K_{nf}$ are respectively the fluid velocity, temperature, Maxwell parameter, heat flux, density, the dynamic viscosity, volumetric thermal expansion coefficient, gravitational acceleration, heat capacitance, thermal conductivity of nanofluids.

Appropriate initial and boundary conditions are

16 $$\begin{aligned}&v(y_{1},0)=0, \ \ \ T(y_{1},0)=T_{\infty }, \ \ \ for \ all \ y_{1}\ge 0, \end{aligned}$$

17 $$\begin{aligned}&v(0,t)=V_{0}H(t), \ \ \ T(0,t)=T_w,\ \ \ t>0, \end{aligned}$$

18 $$\begin{aligned}&v(\infty ,t) \rightarrow 0, \ \ \ T(\infty ,t)\rightarrow T_{\infty }, \,\,\,\,\,\,\,\,\,\,t>0. \end{aligned}$$

where H(t) is a Heaviside unit step function.

Thermo-physical properties are defined in^59^ as follows:

$$\begin{aligned}&\frac{\rho _{nf} - \phi \rho _{s}}{(1-\phi )_{\rho f}} = 1,\ \ \frac{\mu _{nf}}{\mu _{f}}(1-\phi )^{2.5} = 1,\\&\frac{(\rho C_{p})_{nf} - \phi (\rho C_{p})_{s}}{(1-\phi )(\rho C_{p})_{f}} = 1,\\&\frac{K_{nf}}{{K}_{f}}= \frac{K_{s}+2K_{f}-2\phi (K_{f}-K_{s})}{K_{s}+2K_{f}+2\phi (K_{f}-K_{s})},\\&\frac{(\rho \beta _{T})_{nf} - \phi (\rho \beta _{T})_{s}}{(1-\phi )(\rho \beta _{T})_{f}} = 1. \end{aligned}$$

where $\phi$ is the nanoparticle volume fraction, $\rho _{f}$, $\rho _{s}$ are the density of the base fluid and nanoparticle, $\beta _{s}$, $\beta _{f}$ are the volumetric coefficients of thermal expansion of nanoparticle and base fluid, $(C_{p})_{s}$, $(C_{p})_{f}$ are the specific heat capacities of nanoparticle and base fluid at constant pressure and . Here $K_{f}$, $K_{s}$ are thermal conductivities of base fluid and nanoparticle. $\mu _f$ and $\nu _f$ are the dynamic and kinematic viscosity for the base fluid.

Presenting non-dimensional variables and functions

19 $$\begin{aligned} y_{1}^{*}=\frac{V_{0}}{\nu _{f}}y_{1}, \ t^{*}=\frac{V_{0}^{2} }{\nu _{f}}t,\ v^{*}=\frac{v}{V_{0}}, \ \psi ^{*}=\frac{T-T_{\infty }}{T_{w}-T_{\infty }}, \end{aligned}$$

into Eqs. (11), (15) and Eqs. (16)–(18) and reducing $'*'$, we have

20 $$\begin{aligned}&b_{0} \left( \frac{\partial v(y_{1}, t)}{\partial t}+ \lambda ^{\alpha } \frac{\partial ^{\alpha +1}v(y_1,t)}{\partial t^{\alpha +1}}\right) = b_{1} \frac{\partial ^{2}v(y_{1},t)}{\partial y_{1}^{2}}+ b_{2} \text {Gr} \left( 1 + \lambda ^{\alpha } \frac{\partial ^{\alpha }}{\partial t^{\alpha }}\right) \psi (y_{1},t), \end{aligned}$$

21 $$\begin{aligned}&\frac{\partial \psi (y_{1},t)}{\partial t} + \lambda ^{\beta } \frac{\partial ^{\beta +1}\psi (y_1,t)}{\partial t^{\beta +1}}=b_5\frac{\partial ^{2}\psi (y_{1},t)}{\partial {y_{1}}^{2}}, \end{aligned}$$

22 $$\begin{aligned}&v(y_{1},0)=0, \ \ \ \psi (y_{1},0)=0, \ \ \forall \ y_{1}\ge 0, \end{aligned}$$

23 $$\begin{aligned}&v(0,t)=H(t),\ \ \ \psi (0,t)=1, \ \ \ t> 0, \end{aligned}$$

24 $$\begin{aligned}&v(y_{1},t)\rightarrow 0, \ \ \ \ \psi (y_{1},t)\rightarrow 0, \ as \ y_{1}\rightarrow \infty ,\ t>0, \end{aligned}$$

where

$$\begin{aligned}&b_0 = \left\{ (1-\phi ) + \phi \frac{\rho _s}{\rho _f}\right\} , \ \ \ b_1 = \left\{ \frac{1}{(1-\phi )^{2.5}}\right\} , \ \ \ b_2 = \left\{ (1-\phi ) + \phi \frac{(\rho \beta _T)_s}{(\rho \beta _T)_f}\right\} , \\&b_3 = \left\{ (1-\phi ) + \phi \frac{(\rho C_p)_s}{(\rho C_p)_f}\right\} , \ \ \ b_4 = \frac{K_nf}{K_f}, \ \ \ b_5 = \frac{b_4}{b_3 \text {Pr}}, \ \ \ c_1 = \frac{b_0}{b_1}, \ \ \ c_2 = \frac{b_1}{b_5} \\&\lambda = \frac{\lambda _1 V_0^2}{\nu _f}, \ \ \ \text {Pr} = \frac{(\mu C_p)_f}{K_f}, \ \ \ \text {Gr} = \frac{g \nu _f (\beta _T)_f (T_w - T_\infty )}{V_0^{3}}, \end{aligned}$$

where $\frac{\partial ^{\alpha }}{\partial t^{\alpha }}$ and $\frac{\partial ^{\beta }}{\partial t^{\beta }}$ represents the constant proportional Caputo fractional derivative, $\alpha$, $\beta$ are fractional parameters defined in^14^, $\text {Pr}$ is the Prandtl number, and $G\text {r}$ is the thermal Grashof number. Taking Laplace transform of Eqs. (20)–(21) and Eqs. (22)–(24), using Eq. (8), we have

25 $$\begin{aligned}&b_0 \left[ 1+ \lambda ^{\alpha } \left\{ \frac{k_{1}(\alpha )}{s}+k_{0}(\alpha )\right\} s^{\alpha } \right] s {\bar{v}}(y_{1},s)=b_{1}\frac{\partial ^{2}\bar{v}(y_{1},s)}{\partial y_{1}^{2}}+b_{2}Gr \left[ 1+ \lambda ^{\alpha } \left\{ \frac{k_{1}(\alpha )}{s}+k_{0}(\alpha )\right\} s^{\alpha } \right] {\bar{\psi }}(y_{1},s), \end{aligned}$$

26 $$\begin{aligned}&\left[ 1+ \lambda ^{\beta } \left\{ \frac{k_{1}(\alpha )}{s}+k_{0}(\alpha )\right\} s^{\beta } \right] s{\bar{\psi }}(y_{1},s)=b_{5}\frac{\partial ^{2}{\bar{\psi }}(y_{1},s) }{\partial y_{1}^{2}}, \end{aligned}$$

27 $$\begin{aligned}&{\bar{\psi }}(y_{1},0)=0,\ \ \ \ {\bar{v}}(y_{1},0)=0, \end{aligned}$$

28 $$\begin{aligned}&{\bar{\psi }}(0,s)=\frac{1}{s},\ \ \ {\bar{v}}(0,s)=\frac{1}{s}, \end{aligned}$$

29 $$\begin{aligned}&{\bar{\psi }}(y_{1},s)=0,\ \ \ {\bar{v}}(y_{1},s)=0, \ as \ \ y_{1}\rightarrow \infty , \end{aligned}$$

where $k_{0}(\alpha )$ and $k_{1}(\alpha )$ are constants $\in$ (0,1).

Solution of Eq. (26) subject to Eqs. (27)$_1$ - (29)$_1$, we have

30 $$\begin{aligned} \bar{\psi }(y_{1},s)=\frac{1}{s}e^{-y_{1}\sqrt{\frac{1}{b_{5}}\left[ 1+\lambda ^{\beta } \left\{ \frac{k_{1}(\alpha )}{s}+k_{0}(\alpha )\right\} s^{\beta }\right] s}}. \end{aligned}$$

The expression appear in Eq. (30) in exponential form is complicated and difficult to obtain analytically, so we express the this form in its equivalent form:

31 $$\begin{aligned} \bar{\psi }(y_{1},s)=\frac{1}{s}+\sum _{p=1}^{\infty }\sum _{q=0}^{\infty }\sum _{r=0}^{\infty }\frac{[-y_{1}]^{p}\lambda ^{\beta q}[k_{1}(\alpha )]^{r} \Gamma \left( \frac{p}{2}+1\right) \Gamma (q+1)}{p!q!r!(\sqrt{b_{5}})^{p} [k_{0}(\alpha )]^{r+q} s^{1+r-\frac{p}{2}+q-{\beta q}} \Gamma \left( \frac{p}{2}+1-q\right) \Gamma (q+1-r)}. \end{aligned}$$

Taking Laplace inverse of Eq. (31), we have

32 $$\begin{aligned} {\psi }(y_{1},t)=1+\sum _{p=1}^{\infty }\sum _{q=0}^{\infty }\sum _{r=0}^{\infty }\frac{[-y_{1}]^{p}\lambda ^{\beta q}[k_{1}(\alpha )]^{r} t^{r-\frac{p}{2}+q-{\beta q}}{\Gamma \left( \frac{p}{2}+1\right) \Gamma (q+1)}}{p!q!r!(\sqrt{b_{5}})^{p} [k_{0}(\alpha )]^{r+q} \Gamma \left( {r-\frac{p}{2}+q-{\beta q}}\right) \Gamma \left( \frac{p}{2}+1-q\right) \Gamma (q+1-r)}. \end{aligned}$$

Solution of Eq. (25) subject to Eqs. (27)$_2$ - (29)$_2$, we have

33 $$\begin{aligned}&{\bar{v}} (y_{1},s) = \frac{1}{s} \ e^{-y_{1}\sqrt{c_1\left[ 1+\lambda ^{\alpha } \left\{ \frac{k_{1}(\alpha )}{s}+k_{o}(\alpha )\right\} s^{\alpha }\right] s}}\nonumber \\&\quad + \frac{b_2 Gr \left[ 1 + \lambda ^{\alpha } \left\{ \frac{K_1(\alpha )}{s} + K_0(\alpha )\right\} s^\alpha \right] }{s\left[ b_0 \left( 1 + \lambda ^{\alpha } \left\{ \frac{K_1(\alpha )}{s} + K_0(\alpha )\right\} s^\alpha \right) s - c_2 \left( 1 + \lambda ^{\beta } \left\{ \frac{K_1(\alpha )}{s} + K_0(\alpha )\right\} s^\beta \right) s\right] } \nonumber \\&\quad \left\{ e^{-y_{1}\sqrt{\frac{1}{b_{5}}\left[ 1+\lambda ^{\beta }\left\{ \frac{k_{1}(\alpha )}{s}+k_{0}(\alpha )\right\} s^{\beta }\right] s}} - e^{-y_{1}\sqrt{c_1\left[ 1+\lambda ^{\alpha } \left\{ \frac{k_{1}(\alpha )}{s}+k_{o}(\alpha )\right\} s^{\alpha }\right] s}} \right\} . \end{aligned}$$

The Eq. (33) can be expressed in series form so that we can easily find its inverse Laplace analytically

34 $$\begin{aligned}&{\bar{v}}(y_{1},s)=\frac{1}{s} + \sum _{i=0}^{\infty }\sum _{j=0}^{\infty }\sum _{k=0}^{\infty }\frac{(-y_{1}\sqrt{c_1})^{i}\lambda ^{j}(K_{1}(\alpha ))^{k}}{i! j! k! (K_{0}(\alpha ))^{k-j}s^{1-\frac{i}{2}-\alpha j + k}} \frac{\Gamma (\frac{i}{2}+1)\Gamma (j+1)}{\Gamma (\frac{i}{2}-j+1)\Gamma (j+1-k)}\nonumber \\&\quad +\frac{b_2 \text {Gr}}{b_0}\sum _{m_1=0}^{\infty }\sum _{m_2=0}^{\infty }\sum _{m_3=0}^{\infty }\sum _{m_4=0}^{\infty }\nonumber \\&\quad \frac{(-y_1)^{m_{1}} (c_2)^{m_{2}}(-\lambda )^{m_{3}}(K_{1}(\alpha ))^{m_{4}}}{m_1! m_3! m_4! (b_{0})^{m_2}(K_{0}(\alpha ))^{m_4-\frac{m_1}{2}-m_2-m_3} s^{2-\frac{\alpha m_1}{2}+(1-\alpha )m_2-\alpha m_3+m_4}} \frac{\Gamma (m_2+m_3) \Gamma (\frac{m_{1}}{2}+m_2+m_3+1)}{ \Gamma (m_3)\Gamma (\frac{m_{1}}{2}+m_2+m_3+1-m_4)}\nonumber \\&\quad -\frac{b_2 \text {Gr}}{b_0}\sum _{n_1=0}^{\infty }\sum _{n_2=0}^{\infty }\sum _{n_3=0}^{\infty }\sum _{n_4=0}^{\infty } \frac{(-y_1 \sqrt{c_{1}})^{n_{1}} (c_2)^{n_{2}}(\lambda )^{n_{3}}(K_{1}(\alpha ))^{n_{4}}}{n_1! n_3! n_4! (b_{0})^{n_2}(K_{0}(\alpha ))^{n_4-n_2-n_3} s^{2-\frac{ n_1}{2}-\alpha n_3+n_4}}  \nonumber \\&\quad \frac{\Gamma (n_2+n_3+1) \Gamma (\frac{n_{1}}{2}-n_2+1)}{ \Gamma (n_2+n_3+1-n_4)\Gamma (\frac{n_{1}}{2}-n_2+1-n_3)}. \end{aligned}$$

Taking Laplace inverse of Eq. (34), we have

35 $$\begin{aligned}&{\bar{v}}(y_{1},t)=1 + \sum _{i=0}^{\infty }\sum _{j=0}^{\infty }\sum _{k=0}^{\infty }\frac{(-y_{1}\sqrt{c_1})^{i}\lambda ^{j}(K_{1}(\alpha ))^{k} \ t^{k-\frac{i}{2}-\alpha j}}{i! j! k! (K_{0}(\alpha ))^{k-j}s^{1-\frac{i}{2}-\alpha j + k}} \frac{\Gamma (\frac{i}{2}+1)\Gamma (j+1)}{\Gamma (\frac{i}{2}-j+1)\Gamma (j+1-k)\Gamma (1-\frac{i}{2}-\alpha j+k)}\nonumber \\&\quad +b_2 \text {Gr} \sum _{m_1=0}^{\infty }\sum _{m_2=0}^{\infty }\sum _{m_3=0}^{\infty }\sum _{m_4=0}^{\infty } \frac{(-y_1)^{m_{1}} (c_2)^{m_{2}}(-\lambda )^{m_{3}}(K_{1}(\alpha ))^{m_{4}} \ t^{1-\frac{\alpha m_{1}}{2}+(1-\alpha )m_2-\alpha m_3 + m_4}}{m_1! m_3! m_4! (b_{0})^{m_2}(K_{0}(\alpha ))^{m_4-\frac{m_1}{2}-m_2-m_3}}\nonumber \\&\quad \frac{\Gamma (m_2+m_3) \Gamma (\frac{m_{1}}{2}+m_2+m_3+1)}{ \Gamma (m_3)\Gamma (\frac{m_{1}}{2}+m_2+m_3+1-m_4) \Gamma (2-\frac{\alpha m_{1}}{2}+(1-\alpha )m_2-\alpha m_3 + m_4)}\nonumber \\&\quad -b_2 \text {Gr} \sum _{n_1=0}^{\infty }\sum _{n_2=0}^{\infty }\sum _{n_3=0}^{\infty }\sum _{n_4=0}^{\infty } \frac{(-y_1 \sqrt{c_{1}})^{n_{1}} (c_2)^{n_{2}}(\lambda )^{n_{3}}(K_{1}(\alpha ))^{n_{4}} \ t^{1 - \frac{n_{1}}{2} - \alpha n_3 + n_4}}{n_1! n_3! n_4! (b_{0})^{n_2}(K_{0}(\alpha ))^{n_4-n_2-n_3} }  \nonumber \\&\quad \frac{\Gamma (n_2+n_3+1) \Gamma (\frac{n_{1}}{2}-n_2+1)}{ \Gamma (n_2+n_3+1-n_4)\Gamma (\frac{n_{1}}{2}-n_2+1-n_3) \Gamma (2 - \frac{n_{1}}{2}-\alpha n_3+n_4)}. \end{aligned}$$

## Numerical outcomes and analysis

This paper deals with the investigation of Clay nanoparticles in free convection of a Maxwell fluid. The analytical solutions satisfies the initial and boundary conditions. The solutions are obtained with the application of novel fractional derivative and Laplace transformation. The influence of nanoparticles as well fractional parameter are discussed through some graphs.

**Figure 2:**
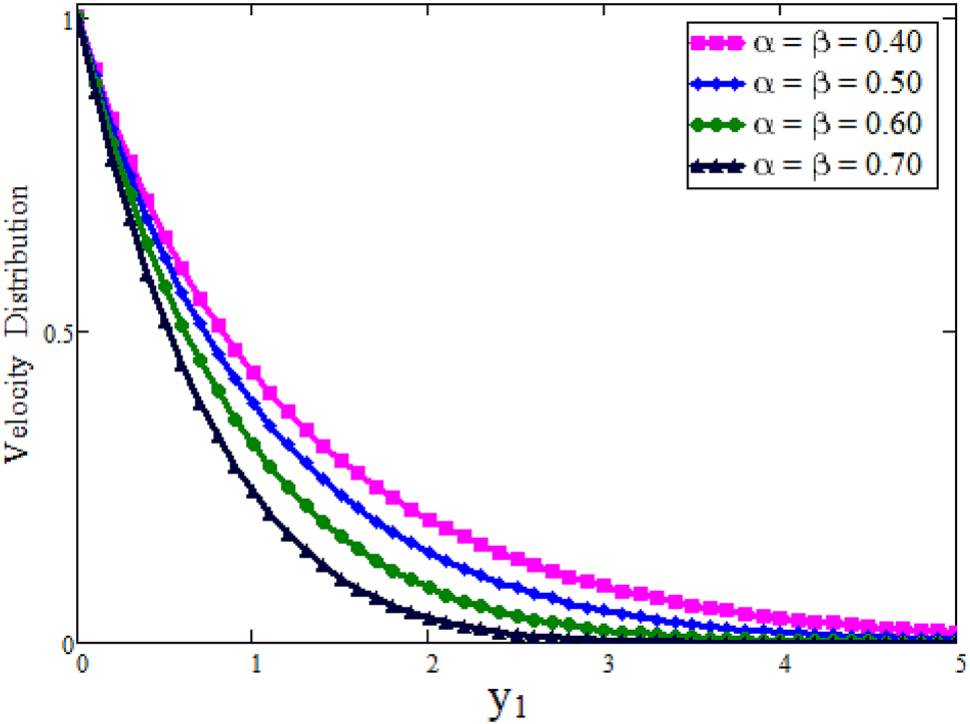
Velocity profile against $y_{1}$ due to $\alpha$ for small time, when: $t=0.01$, $\phi =0.01$, $\text {Pr}=6.2$, $\text {Gr}=0.1$ and $\lambda =0.5.$

**Figure 3:**
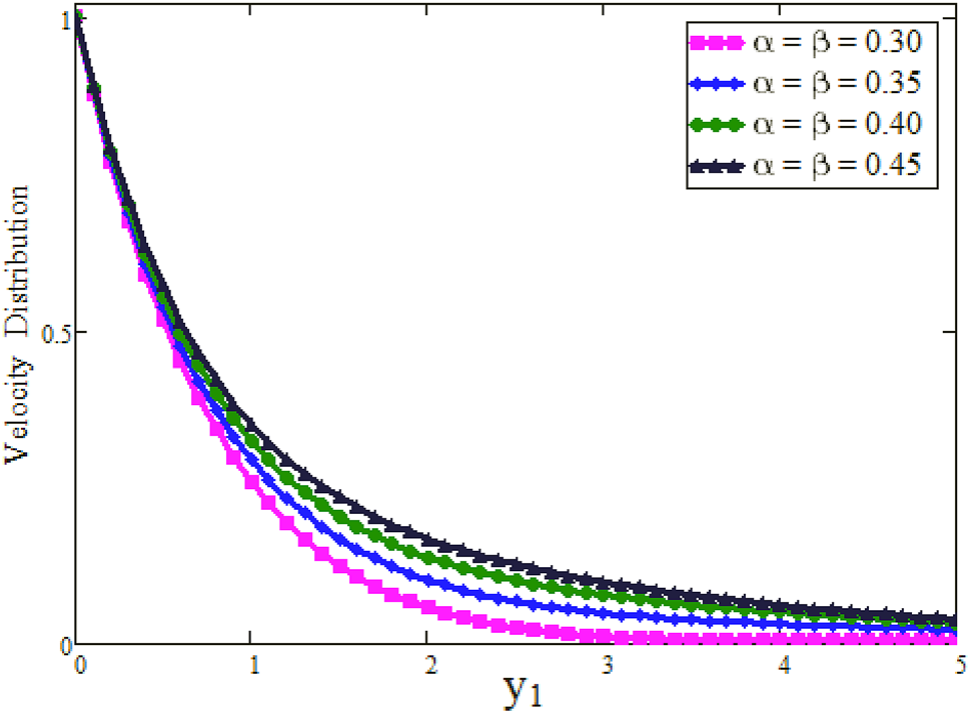
Velocity profile against $y_{1}$ due to $\alpha$ for large time, when: $t=0.1$, $\phi =0.01$, $\text {Pr}=6.2$, $\text {Gr}=0.1$ and $\lambda =0.5.$

**Figure 4:**
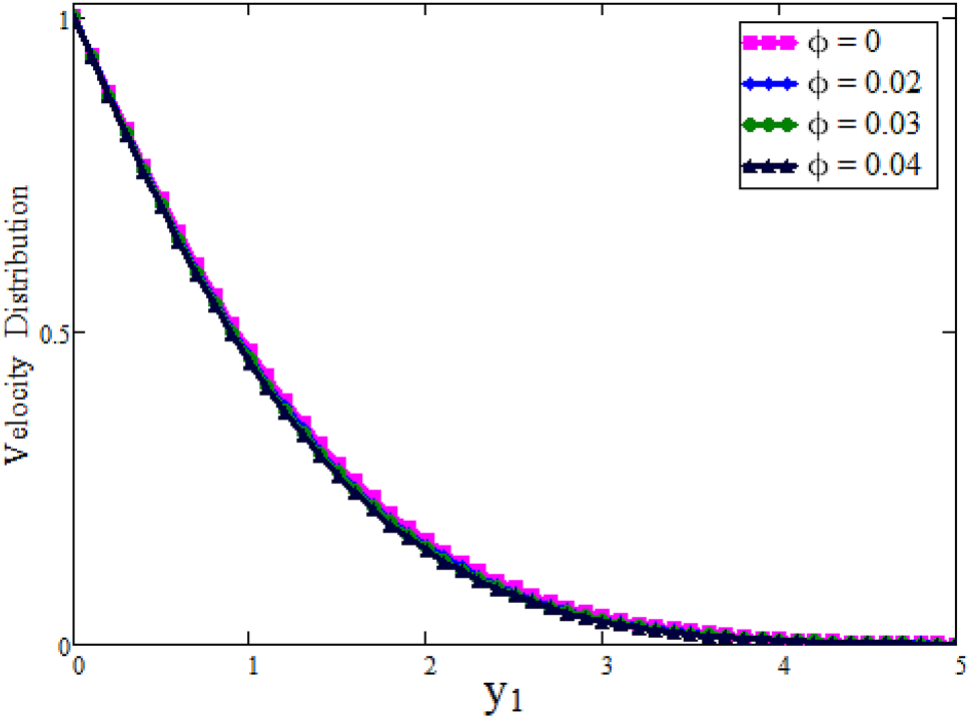
Velocity profile against $y_{1}$ due to $\phi$, when: $t=0.5$, $\text {Gr}=0.1$, $\text {Pr}=6.2$, $\lambda =0.01$ and $\alpha =0.5.$

**Figure 5:**
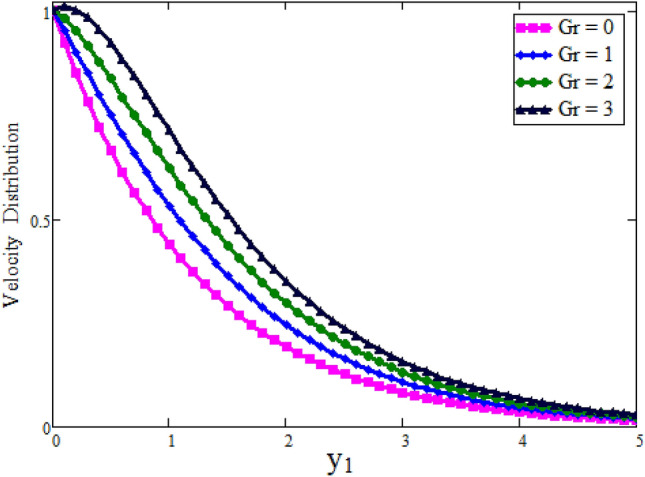
Velocity profile against $y_{1}$ due to $\text {Gr}$, when: $t=0.25$, $\phi =0.04$, $\text {Pr}=6.2$, $\alpha =0.2$ and $\lambda =0.01$.

**Figure 6:**
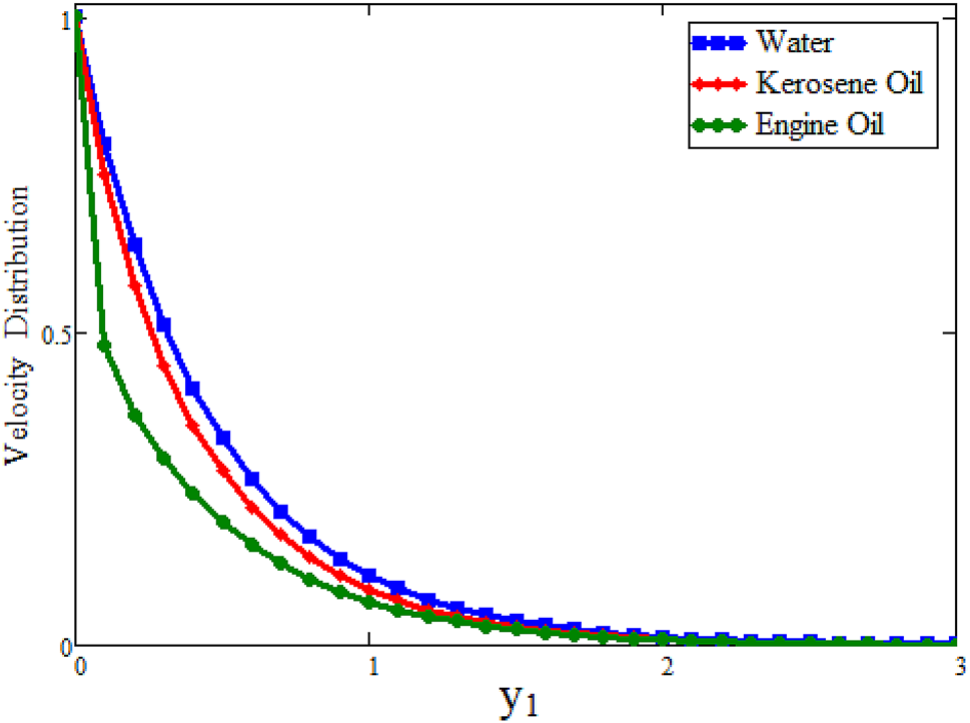
Comparison of velocity profile with different base fluids, when: $t=0.005$, $\phi =0.42$, $\text {Gr}=5$, $\alpha =0.1$ and $\lambda =0.51.$

Figure 2 depicted to see the variation of fractional parameter $\alpha$. The maximum decay in velocity can be obtained for small time. It is clear from the Fig. 2 by increasing values of $\alpha$ velocity exhibits the maximum decay for small time. This behavior can be reversed for large values of time is shown in Fig. 3. Further, fractional parameter can be used to control the momentum boundary layer thickness. The relationship of velocity of clay nanoparticles and volume fraction $\phi$ is discussed in Fig. 4. For large values of $\phi$ fluid velocity will decrease as viscous forces became stronger with increasing $\phi$. Figure 5 high lights the influence of Grashof number $\text {Gr}$. Fluid velocity increases as we enhance the value of $\text {Gr}$. $\text {Gr}$ describes the influence of the thermal buoyancy force to the viscous force. If $\text {Gr}$ equal to zero, then there is no free convection current, if $\text {Gr}$ is greater than zero, plate is outwardly chilled and if $\text {Gr}$ is less than zero, plate is outside frenzied. It means larger plate is outwardly chilled increasing the velocity and effect is reverse of $\text {Pr}$. Figure 6 represents the contrast of velocity profile for three different types of base fluids (water, kerosene, and engine oil). It is observed that the velocity of water-based clay nanofluid fluid is larger than kerosene oil and engine oil-based clay nanofluids, respectively. Because the thermal conductivity of water is larger than that of kerosene oil and engine oil then the velocity of water-based clay nanofluid is larger than the others.

**Figure 7:**
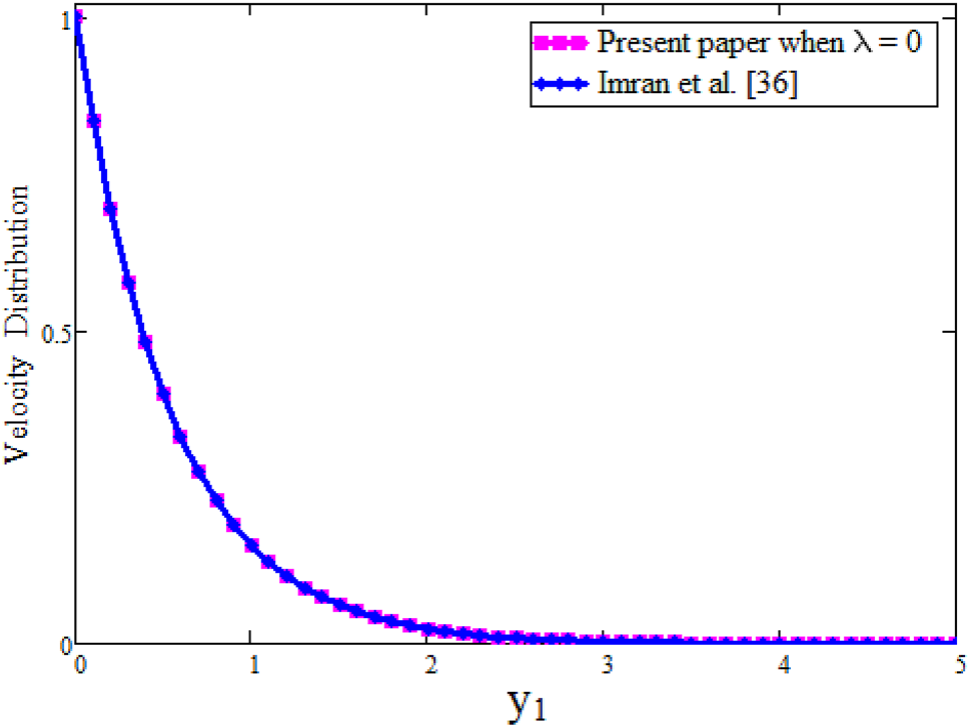
Comparison of velocity profile when $\lambda =0$ with Imran et al.^61^, when: $t=0.1$, $\phi =0.04$, $\text {Pr}=6.2$, $\text {Gr}=0.1$ and $\alpha =0.2.$

**Figure 8:**
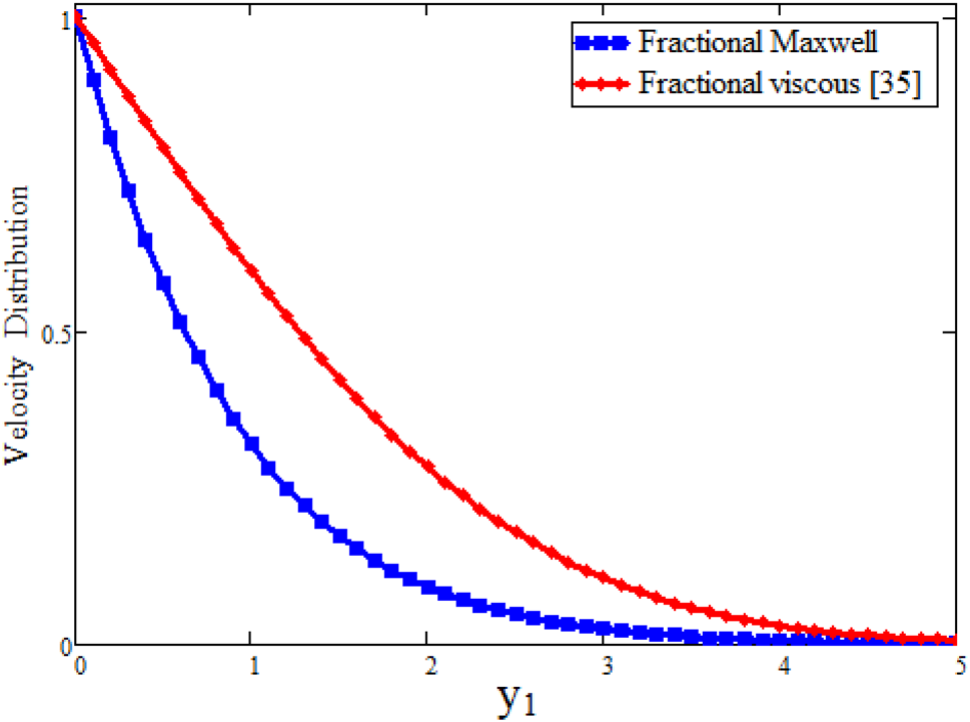
Comparison of velocity profile of fractional Maxwell fluid with viscous fluid^59^, when: $t=1.5$, $\phi =0.4$, $\text {Pr}=6.2$, $\lambda =0.1$, $\text {Gr}=0.1$ and $\alpha =0.8.$

**Figure 9:**
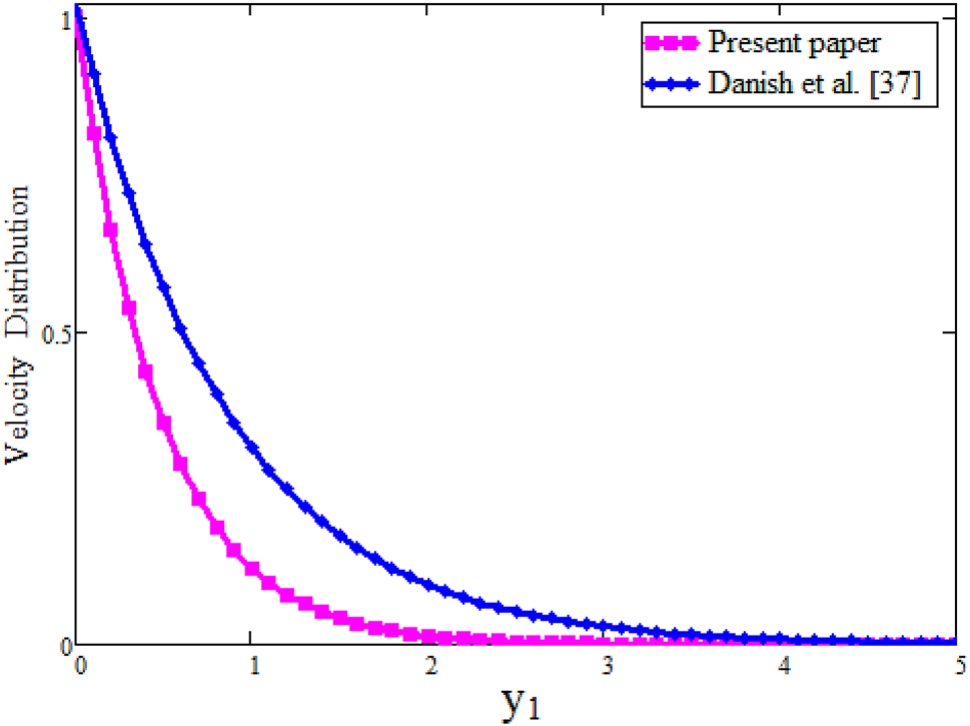
Comparison of velocity profile with Danish et al.^69^, when: $t=0.02$, $\phi =0.04$, $\text {Pr}=6.2$, $\lambda =0.001$, $\text {Gr}=0.1$, $M=0.1$ and $\alpha =0.2.$

**Figure 10:**
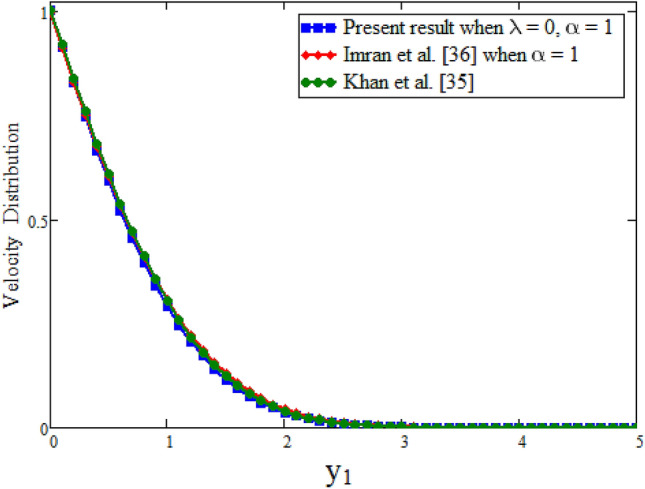
Comparison of velocity profile with Khan et al.^59^ and Imran et al.^61^, when: $t=0.095$, $\phi =0.72$, $\text {Pr}=6.2$ and $\text {Gr}=0.1.$

Figure 7 our obtained results by taking $\lambda$ = 0 are compared with the results of Imran et al.^61^ while keeping other parametric values constant. Table 2 represents the velocity comparison of present paper when $\lambda$ = 0 and Imran et al.^61^ for different values of parameter $\alpha$. We have seen that both Fig. 7 and Table 2 are in good agreement with each other. Figure 8 is plotted to see the velocity comparison of hybrid fractional derivative and Khan et al.^59^. Since the velocity obtained in^59^ is for viscous fluid and in the present study for Maxwell fluid. This figure shows the viscous fluid is swiftest than Maxwell fluid. The reason is that Maxwell fluid is non-Newtonian one and more thicker than viscous. Table 3 shows the velocity comparison of current paper and Khan et al.^59^ for different values of parameter $\alpha$. In both cases we have found that velocity is smaller for CPC fractional model. Physically, fractional operator is responsible for the history of the model and can have better control for momentum boundary layer. Figure 9 shows the velocity comparison between present result and Danish et al.^69^ with the solutions obtained with the same fractional operator for viscous fluid and we see that velocity is smaller for constant proportional Caputo model of Maxwell fluid over viscous fluid. Due to less viscosity of viscous fluid, the it flows faster than Maxwell fluid. The velocity contrast of present result and Danish et al.^69^ is shown in Table 4 and velocity is minimum for constant proportional Caputo model. The velocity comparison between present result when $\lambda$ =0, $\alpha =1$, Imran et al.^61^ when $\alpha$ = 1 and Khan et al.^59^ is shown in Fig. 10. We see that these results are in good agreement. Table 5 represents the velocity contrast of present result and published results and we see that results are same. The influence of fractional parameter $\beta$ on Nusselt number is studied numerically in Table 6. Nusselt number is decreasing function of fractional parameter $\beta$. The influence of fractional parameter $\alpha$ on Skin friction is evaluated numerically in Table 7. Skin friction is decreasing function of fractional parameter $\alpha$.

**Table 2:** Effect of fractional parameter on dimensionless velocity.

$y_1$	$\alpha =0.99$	$\alpha =0.99$
	Present paper when $\lambda =0$	Imran et al.^61^
0.0	1	1
0.1	0.942	0.941
0.2	0.885	0.882
0.3	0.828	0.824
0.4	0.772	0.767
0.5	0.717	0.712
0.6	0.664	0.658
0.7	0.612	0.606
0.8	0.562	0.556
0.9	0.514	0.509
1.0	0.468	0.463

**Table 3:** Effect of fractional parameter on dimensionless velocity.

$y_1$	$\alpha =0.4$	Viscous^59^	$\alpha =0.7$	Viscous^59^	$\alpha =0.9$	Viscous^59^
	Fractional Maxwell		Fractional Maxwell		Fractional Maxwell	
0.0	1	1	1	1	1	1
0.1	0.939	0.958	0.946	0.958	0.950	0.958
0.2	0.880	0.916	0.893	0.916	0.901	0.916
0.3	0.822	0.874	0.840	0.874	0.851	0.874
0.4	0.766	0.833	0.787	0.833	0.802	0.833
0.5	0.711	0.791	0.736	0.791	0.753	0.791
0.6	0.659	0.751	0.686	0.751	0.705	0.751
0.7	0.608	0.710	0.637	0.710	0.658	0.710
0.8	0.560	0.671	0.590	0.671	0.611	0.671
0.9	0.514	0.632	0.543	0.632	0.566	0.632
1.0	0.470	0.594	0.499	0.594	0.521	0.594

**Table 4:** Effect of fractional parameter on dimensionless velocity.

$y_1$	$\alpha =0.2$	$\alpha =0.2$	$\alpha =0.5$	$\alpha =0.5$
	Present paper	Danish et al.^69^	Present paper	Danish et al.^69^
0.0	1	0.987	1	0.991
0.1	0.870	0.881	0.870	0.873
0.2	0.744	0.785	0.743	0.769
0.3	0.623	0.700	0.623	0.677
0.4	0.512	0.625	0.511	0.595
0.5	0.412	0.557	0.411	0.524
0.6	0.325	0.496	0.324	0.460
0.7	0.252	0.442	0.250	0.405
0.8	0.191	0.394	0.189	0.355
0.9	0.142	0.351	0.141	0.312
1.0	0.104	0.312	0.102	0.274

**Table 5:** Comparisons of dimensionless velocity.

$y_1$	Present result	Imran et al.^61^	Khan et al.^59^
	When $\lambda = 0, \alpha = 1$	When $\alpha$ = 1	
0.0	1	0.998	1
0.1	0.944	0.939	0.944
0.2	0.888	0.882	0.888
0.3	0.833	0.825	0.833
0.4	0.778	0.771	0.778
0.5	0.725	0.717	0.725
0.6	0.673	0.666	0.673
0.7	0.622	0.616	0.622
0.8	0.573	0.568	0.573
0.9	0.526	0.523	0.526
1.0	0.481	0.479	0.481

**Table 6:** Statistically analysis of Nusselt number for the effect of fractional parameter.

$\beta$	Nu	Nu	Nu
	t = 2	t = 3.5	t = 5
0.1	1.908	1.767	1.708
0.2	1.855	1.677	1.592
0.3	1.797	1.587	1.480
0.4	1.735	1.498	1.374
0.5	1.668	1.411	1.272
0.6	1.598	1.325	1.177
0.7	1.525	1.242	1.087
0.8	1.450	1.161	1.004
0.9	1.374	1.084	0.926
1.0	1.297	1.011	0.856

**Table 7:** Statistically analysis of Skin friction for the effect of fractional parameter.

$\alpha$	$C_{f}$	$C_{f}$	$C_{f}$
	t = 1	t = 2	t = 3.5
0.1	1.066	1.437	− 61.378
0.2	1.049	1.413	− 63.535
0.3	1.030	1.388	− 65.760
0.4	1.007	1.363	− 68.057
0.5	0.981	1.338	− 70.427
0.6	0.954	1.313	− 72.873
0.7	0.923	1.287	− 75.399
0.8	0.890	1.262	− 78.007
0.9	0.855	1.236	− 80.699
1.0	0.819	1.211	− 83.479

## Conclusions

The convection heat transfer in clay nanofluid using Maxwell model is studied. Exact solutions for velocity and temperature are evaluated with help of the Laplace transform technique. We have drawn the comparisons with the published results and they are in good agreement. Key findings of current study are:

1. Velocity of drilling fluid, for small values of time shows decay behavior for increasing fractional parameter and concentration of nanoparticles.
2. Water based drilling nanofluids exhibit maximum velocity rather than oil based drilling fluids.
3. Different Comparisons of present result of velocity are drawn with Khan et al.^59^, Imran et al.^61^ and Danish et al.^69^.
